# Author response to: The impact of virtual-reality simulation training on operative performance in laparoscopic cholecystectomy: meta-analysis of randomized clinical trials

**DOI:** 10.1093/bjsopen/zrac136

**Published:** 2022-11-22

**Authors:** Gemma Humm, Helen Mohan, Christina Fleming, Rhiannon Harries, Christopher Wood, Khaled Dawas, Danail Stoyanov, Laurence B Lovat

**Affiliations:** Wellcome/Engineering and Physical Sciences Research Council Centre for Interventional and Surgical Sciences, University College London, London, UK; UCL Division of Surgery and Interventional Science, University College London, London, UK; Department of General and Colorectal Surgery, Peter MacCallum Cancer Centre, Melbourne, Victoria, Australia; Department of General and Colorectal Surgery, University Hospital Limerick, Limerick, Ireland; Department of General and Colorectal Surgery, Swansea Bay University Health Board, Swansea, UK; Department of General and Colorectal Surgery, University College London Hospitals NHS Foundation Trust, London, UK; UCL Division of Surgery and Interventional Science, University College London, London, UK; Wellcome/Engineering and Physical Sciences Research Council Centre for Interventional and Surgical Sciences, University College London, London, UK; Wellcome/Engineering and Physical Sciences Research Council Centre for Interventional and Surgical Sciences, University College London, London, UK; UCL Division of Surgery and Interventional Science, University College London, London, UK

*Dear Editor*

We thank Toale *et al.* for their correspondence on ‘The impact of virtual-reality simulation training on operative performance in laparoscopic cholecystectomy: meta-analysis of randomized clinical trials’^1^ and welcome the opportunity to respond.

This correspondence has highlighted an important distinction on the methodology of some of the included studies. Four of the included studies trained their participants to a defined proficiency using virtual-reality training (VRT) and compared performance with participants who received no additional training (NAT)^2–5^. Individually these studies favoured VRT, which would likely be confirmed by meta-analysis. We acknowledge that inclusion of train-to-proficiency model subgroup analysis could be beneficial in demonstrating a positive relationship between VRT and performance and should be considered for future work. Of the three studies identified in the correspondence, two justified the training duration with previous evidence, but it is reasonable to question whether sufficient time was available for all participants to all achieve a defined level of competence, particularly with mixed-ability participants. Most available and included studies use the train-to-time model, which is likely a reflection of the training delivered of the time. With the implementation of the new competency-based general surgery curriculum in the UK and Ireland, it is likely that the train-to-proficiency model methodology and barriers to the implementation of simulation training will be more widely reflected in academic research.

Ideally, all participants would have comparable baseline characteristics and proficiency before intervention. Meta-analysis of studies with heterogenous participants (such as medical students *versus* surgical trainees) can influence results. This can also serve to reflect the heterogenous levels of skills, experience, and learning curves of those in a cohort of junior trainees, which should be considered in the interpretation of results.

## References

[1] Humm G, Mohan H, Fleming C, Harries R, Wood C, Dawas K et al The impact of virtual reality simulation training on operative performance in laparoscopic cholecystectomy: meta-analysis of randomized clinical trials. BJS open 2022;6:zrac086 10.1093/bjsopen/zrac086 PMC9291386 35849132

[2] Aggarwal R, Ward J, Balasundaram I, Sains P, Athanasiou T, Darzi A. Proving the effectiveness of virtual reality simulation for training in laparoscopic surgery. Ann Surg 2007;246:771–779 17968168 10.1097/SLA.0b013e3180f61b09

[3] Ahlberg G, Enochsson L, Gallagher AG, Hedman L, Hogman C, McClusky DA et al Proficiency-based virtual reality training significantly reduces the error rate for residents during their first 10 laparoscopic cholecystectomies. Am J Surg 2007;193:797–804 17512301 10.1016/j.amjsurg.2006.06.050

[4] Palter VN, Orzech N, Reznick RK, Grantcharov TP. Validation of a structured training and assessment curriculum for technical skill acquisition in minimally invasive surgery: a randomized controlled trial. Ann Surg 2013;257:224–230 23013806 10.1097/SLA.0b013e31827051cd

[5] Våpenstad C, Hofstad EF, Bø LE, Kuhry E, Johnsen G, Mårvik R et al Lack of transfer of skills after virtual reality simulator training with haptic feedback. Minim Invasive Ther Allied Technol 2017;26:346–354 28486087 10.1080/13645706.2017.1319866

